# Supplementary material for: Impact of institutional support and green knowledge transfer on university students’ absorptive capacity and green entrepreneurial behavior: The moderating role of environmental responsibility
Source: PLoS One. 2024 May 21;19(5):e0304070. doi: 10.1371/journal.pone.0304070 (PMC11108129; doi:10.1371/journal.pone.0304070)
Supplement: Attachment — Submitted filename: Response to Reviewers.docx [file pone.0304070.s001.docx]

**Reviewer #1: Impact of institutional support and green knowledge transfer on university students’ absorptive capacity and green entrepreneurial behavior: the moderating role of environmental responsibility**

**Thank you for granting me the opportunity for review. Very interesting and new insight in the way of scientific research on the role of environmental responsibility especially in the emerging nations. Sufficient literature is applied, and the research gap is articulated in a good way. Methodological tools have been adequately applied. The theme of the paper is very interesting and has a lot of practical implications. Overall, the paper is well-written and can be considered for publication. However, the following are some minor suggestions to improve the paper.**

**• Abstract should be improved in the following way. First, you follow the aims/Objective. Second, you should follow the methodology. Third, you should follow the findings and conclusion drawn from their findings. Fourth implications.**

**• Introduction should be developed scientifically. First, you discuss the problems and their significance. The clear objectives and questions should be mentioned. The contribution of the study should be mentioned clearly,**

**• There are a lot of grammatical mistakes, authors need to improve.**

**• Justified your findings with previous literature that discussed in the literature section.**

**Best of Luck**

**Response**

Thank you for your constructive feedback and encouraging comments on our manuscript. I have made the following revisions in accordance with your suggestions:

1. I have restructured the abstract to sequentially include the aims/objectives, methodology, findings, and implications.
2. The introduction has been scientifically developed to more clearly articulate the problem, its significance, and the study's specific objectives and contributions.
3. I have thoroughly reviewed the manuscript and corrected grammatical errors to improve the overall quality of the writing.
4. The findings have been more explicitly justified and linked with the existing literature discussed in the literature review section.

**Reviewer #2: First of all, I appreciate the author contribution towards novelty. The paper is well written but I have few suggestions for authors as per my expertise.**

**1) abbreviation used in abstract portion that is not meaningful, you can explain them first and don't use any abbreviation in abstract portion specifically.**

**2) The literature review segment till analysis is perfectly written but conclusion portion is missing.**

**3) Specify more literature about green entrepreneurship.**

**Response**

I greatly appreciate your positive remarks and valuable suggestions. In line with your recommendations:

1. I have revised the abstract to explain the key terms initially and avoided the use of abbreviations, thereby making it more accessible and understandable.
2. A conclusion section has been added to effectively summarize the key findings and their implications.
3. More literature on green entrepreneurship has been incorporated to enrich the theoretical underpinnings of the study.

I believe these revisions have significantly enhanced the quality of our manuscript and thank you for your insightful guidance in this process.

Top of Form
